# Study on the effectiveness and safety of Xingpi Yanger granule combined with Saccharomyces boulardii for rotavirus enteritis in children: A protocol for systematic review and meta-analysis

**DOI:** 10.1097/MD.0000000000025593

**Published:** 2021-04-23

**Authors:** Cheng-Ying Qiu, Zao-Xia Guo, Gui-Hua Zhang, Yong-Hong Feng, Ying-Yun Deng, Xian-Jia Chen, Xiao-Dong Wu, Shan-Wen Huang

**Affiliations:** a Department of Pediatrics, Sanya People 's Hospital, No. 558 Jiefang Road, Tianya District, Sanya City; b Department of Internal Medicine of Traditional Chinese Medicine, Danzhou Hospital of Traditional Chinese Medicine, No. 30–20 Zhongxing Avenue, Danzhou City; c Department of Neonatology, Haikou Hospital of the Matemal and Child Health, NO.6 Wentan Road, Guoxing Avenue, Qiongshan District, Haikou City; d Department of Spleen-Stomach Diseases, Danzhou Hospital of Traditional Chinese Medicine, No. 30-20 Zhongxing Avenue, Danzhou City; e Department of Geriatrics, Danzhou Hospital of Traditional Chinese Medicine, No. 30-20 Zhongxing Avenue, Danzhou City; f Department of Pediatrics, Haikou Hospital of the Matemal and Child Health, NO.6 Wentan Road, Guoxing Avenue, Qiongshan District, Haikou City, Hainan Province, China.

**Keywords:** meta-analysis, protocol, rotavirus enteritis in children, Saccharomyces boulardii, xingpi yanger granule

## Abstract

**Background:**

To systematically evaluate the effectiveness and safety of traditional Chinese medicine preparation XPYEG combined with SBI and SBI alone in the treatment of REC, and to provide the reference in drugs for the clinical treatment of children with rotavirus enteritis.

**Methods:**

Retrieving the English databases: PubMed, Cochrane Library and Embase; Chinese databases: CNKI, CBM and WANFANG Data. Retrieving a randomized controlled trial of XPYEG and SBI in the treatment of REC. The retrieval time is from the above database until September 2020. The retrieval strategy of combining free words and subject words is adopted, and the references included in the literature are searched manually in accordance with the literature studied in this paper and not included in the above database. Two researchers screen the literature according to the literature inclusion and exclusion criteria, extract valid data and evaluate the quality of the literature, and cross-check it. Using the RevMan 5.3 software to conduct the meta-analysis on the main outcome and secondary outcome indicators of the included literature, while assessing the evidence quality of included study.

**Results:**

The effectiveness and safety of XPYEG and SBI in the treatment of REC are presented through the main and secondary outcome indicators.

**OSF Registration number:**

DOI 10.17605/OSF.IO/3QSZG.

**Conclusion:**

This study will conclude whether the combination of XPYEG and SBI is more effective than SBI alone in the treatment of REC, and whether the medication increases the risk of adverse reactions compared with single medication.

**Ethics and dissemination:**

This study does not involve the specific patients, and all research data comes from publicly available professional literature, so an ethics committee is not required to conduct an ethical review and approval of the study.

## 1 Introduction

Rotavirus (RV) was named in 1975 for its wheel-like form, which was first discovered and reported by Australian scholar bishop in 1973 in duodenal epithelial cells of children with acute diarrhea.^[1]^ Before the discovery of the RV, it had been realized that acute diarrhea had a “non-bacterial” pathogen, but it was difficult to isolate the imaginary “virus” by means of bacterial culture. Until bishop discovered and reported rotavirus, it was found that rotavirus was the most common pathogen causing diarrhea in children.^[2]^ Rotavirus enteritis in children (REC) is an acute infectious and self-limiting disease caused by rotavirus infection, with the highest incidence in children between the 6 months and 2 years of age. According to the U.S. Centers for Disease Control and Prevention about 140 million children worldwide are infected with the disease and about 1 million people dies each year.^[3]^ The incidence rate in developing countries is higher than in developed countries. The disease can occur all year round, but it occurs mostly in autumn and winter, and shows the epidemic onset patterns. From September to December in China is the peak of the disease, and there is also a clear trend of epidemic incidence, during which the detection rate of the virus can be as high as 90%.^[4]^

The disease is mainly transmitted through the respiratory tract, digestive tract, the source of infection is patients and the virus carriers. The virus transmits into the gastrointestinal tract through the mouth, incubation period is generally 2 days. The intestinal tract begins to excrete the virus on the day before symptoms appear. The 3–5 days of the disease course is the peak to excrete the virus. the process generally lasts about 7 days, and a few patients can last 2 weeks.^[5]^ The characteristics of the disease are: acute illness, accompanied by fever and upper respiratory tract infection symptoms. The initial stage of the disease can occur vomiting, followed by diarrhea, stools with many times, more quantity, more moisture, yellow water or egg-like stool and with a small amount of mucus, more odorless and so on, the disease often causes convulsions, heart muscle damage and other complications.^[6]^

So far, the etiology and pathogenesis of REC have been basically clarified. Rotavirus invades the epithelial cells of duodenum and jejunum, resulting in the absorption dysfunction of intestinal microvilli and the decrease of disaccharidase activity, finally leads to unsorted intestinal fluid and indigestion of sugars in food in the intestinal cavity, the osmotic pressure of secondary bowel fluid increases and micro-fluffy cortide cell sodium transport functional disorders, resulting in a large number of water-like stools.^[7]^ However, recent clinical studies have found that children with rotavirus enteritis can also be affected by sex, age, onset season, feeding methods and self-body mass, immune function due to the physiological and pathological characteristics of children themselves.^[8]^ At present, there is no special treatment for rotavirus enteritis, the commonly used treatment methods are gastrointestinal mucosa protection agents, antiviral therapy, micro-ecological therapy, rehydration therapy to maintain hydrolyte balance and acid-base balance, traditional Chinese medicine treatment, treatment and support treatment etc.^[9,10]^ Traditional Chinese medicine attributes children's rotavirus enteritis to the category of “diarrhea”, and believes that the occurrence of the disease is related to factors such as improper feeding, improper diet, and external cold and cold. Under the influence of a combination of factors, spleen and gastric transport disorders, water moisture evil gas does not follow the usual way out. In recent years, traditional Chinese medicine have made remarkable progress in the treatment of REC. Many studies have been published on the good clinical effectiveness of traditional Chinese medicine in the treatment of REC.^[11]^

However, there is still no high-quality evidence on the combined use of Chinese medicine or Chinese and Western medicine in the treatment of REC. Therefore, this study uses the method of systematic evaluation, combines with the respective advantages of Western medicine and Traditional Chinese medicine, to study and compare Xingpi Yanger granule (XPYEG) and Saccharomyces boulardii (SBI), the two common drugs used in the treatment of REC, and to analyze the advantages and disadvantages and safety of combined drug use over a single drug use.^[12]^ It provides a reliable scientific basis for clinical treatment of the disease, so as to find new methods and ways to treat REC.

## 2 Methods

### 2.1 Registration and reporting

The study protocol has been registered on the OSF platform, registration number: DOI 10.17605/OSF.IO/3QSZG (https://osf.io/3qszg).

### 2.2 Ethics and dissemination

This study does not involve the specific patients, and all research data comes from publicly available professional literature, so an ethics committee is not required to conduct an ethical review and approval of the study.

### 2.3 Eligibility criteria

#### 2.3.1 Types of studies

Through the network electronic database and manual access to literature, a wide range of randomized controlled trials on XPYEG combined with SBI in the treatment of REC are widely collected. Whether the random method is correct or not, the language of literature retrieval is Chinese and English.

#### 2.3.2 Types of patients

Children who are clinically and laboratory diagnosed with rotavirus enteritis are selected as the subjects of this experiment.

Diagnostic criteria: According to the *Expert Consensus on the Principles of Diagnosis and Treatment of Diarrhea Diseases in Children* on the content of “rotavirus enteritis””, formulating the Western medical diagnostic standards for rotavirus enteritis, in which the diagnosis needs to have clinical diagnosis and viral pathogen diagnosis basis.^[13]^

(1) The stools are egg-like or watery, with varying amounts, and the number of stools is more than 5 times per day;
(2) It may be accompanied by fever, vomiting, loss of appetite, dehydration and other;
(3) Laboratory examination: under the stool microscope to see a large number of fat balls, or a small number of white blood cells, red blood cells. Human rotavirus antigen test: positive.

#### 2.3.3 Inclusion criteria

(1) It conforms to the diagnostic criteria of REC.
(2) Disease course: 6 to 24 h, age: 6 months to 2 years old.
(3) No medication has been used before the treatment, including laxatives, intestinal probiotics, intestinal mucous membrane protection and antiviral drugs.
(4) There is good compliance with clinical observation and evaluation of clinical effectiveness.

#### 2.3.4 Exclusion criteria

(1) People with underlying or combined other diseases, such as immunodeficiency diseases, malnutrition, gastrovascular malformations, and congenital heart disease.
(2) Severe dehydration, severe electrolyte disorder and severe acidosis.
(3) Severe vomiting, it may affect the absorption of drugs and determine the therapeutic effect.
(4) Those who fail to take other anti-laxative drugs and incomplete information in accordance with the standard medication and treatment.
(5) Non-randomized controlled trials (review literature, repetitive published clinical trial literature and animal trial research literature).
(6) Incomplete literature for data reporting.

### 2.4 Interventions

Intervention method of the experimental group: XPYEG combined with SBI combined with basic treatment.

Intervention method of the control group: SBI combined with basic treatment.

The specifications of drugs, the use dose and course of treatment are not limited, this situation is likely to lead to a greater bias in the outcome indicators, but this study will use the method of subgroup analysis to analyze these factors.

Basic treatment: adjusting the diet, correcting the dehydration and acid-base balance disorders, etc., to give appropriate treatment for complications.

### 2.5 Outcome indicators

#### 2.5.1 The primary outcomes

(1) Overall efficiency. The standards shall be formulated in the “Trial Implementation of guiding principles for clinical research of new Chinese medicine”” formulated and promulgated by the Ministry of health of the people's Republic of China.
  - Cure: after the treatment of 72 h, abdominal pain, vomiting symptoms disappear, the stool characteristics and frequency are normal.
  - Effective: after the treatment of 72 h, abdominal pain, vomiting symptoms disappear, the stool characteristics and frequency significantly improved.
  - Invalid: the disease is aggravated, abdominal pain, vomiting symptoms are not improved or aggravated, the stool characteristics and frequency is not significantly reduced or increased. Total efficiency (number of cures and effective cases) / Total number of cases × 100%.
(2) Negative rate of rotavirus antigen.
(3) The time of the child's fever abatement.
(4) The patient's antidiarrheal time.
(5) Dehydration of the child.

#### 2.5.2 The secondary outcomes

(1) The disappearance time of vomiting symptoms.
(2) The disappearance time of abdominal pain symptoms.
(3) Myocardial enzyme spectrum level: creatine phosphokinase (CPK), creatine kinase, MB form (CK-MB), aspartate aminotransferase (AST), lactate dehydrogenase (LDH).
(4) Length of stay.
(5) Inflammatory factor levels.
(6) The occurrence of adverse reactions.

### 2.6 Retrieval strategy

Using the computer to retrieve the PubMed, Cochrane Library, Embase, WANFANG Data, CNKI, CBM and other Chinese and English literature databases, collecting the literatures on the clinical effectiveness and safety of XPYEG combined with SBI alone in the treatment of REC, and by means of standards for inclusion in the study, manually retrieving references to the relevant literature. The time limit is from the establishment of the database to September 2020, the retrieval language is Chinese and English, the Chinese retrieval words include: “Xing-pi-yang-er-ke-li”, “Bu-la-shi-jiao-mu-jun”, “Xiao-er-lun-zhuang-bing-du-chang-yan”, and so on. The English retrieval words include: “Xingpi Yanger granule”, “saccharomyces boulardii”, “rotavirus enteritis in children”, and so on. The retrieval strategy for literature can be found in Table 1.

**Table 1:** The results of retrieval strategy in Cochrane Library.

Number	Search items
#1	(Xingpi Yanger granule):ti,ab,kw
#2	(Saccharomyces boulardii OR Saccharomyces boulardius OR boulardii, Saccharomyces OR boulardius, Saccharomyces):ti,ab,kw
#3	(rotavirus infections OR infection, rotavirus OR infections, rotavirus OR rotavirus infection OR rotavirus OR rotaviruses OR neonatal calf diarrhea virus):ti,ab,kw
#4	(child OR children):ti,ab,kw
#5	(enteritis OR enteritides):ti,ab,kw
#6	#3 AND #4 AND #5
#7	#1 AND #2 AND #6

### 2.7 Data extraction and management

**Figure 1:**
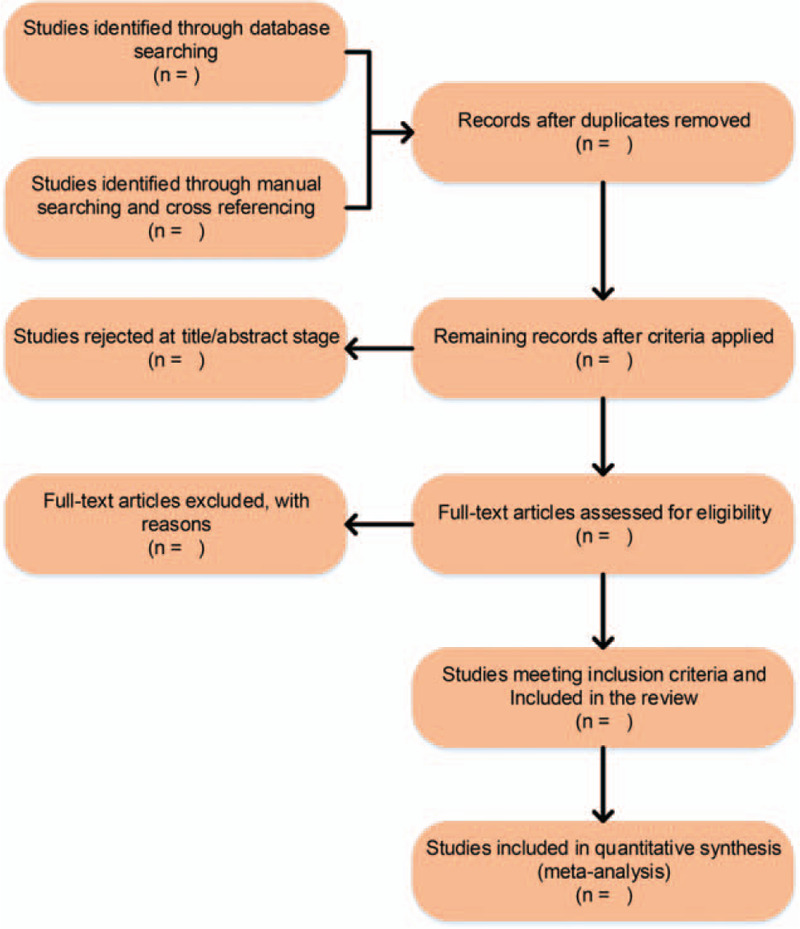
The PRISMA flow chart of literature selection.

The two researchers independently carry out the literature screening. After retrieving the major databases by computer, all the filtered literature titles and abstracts are imported into the literature management software, duplicate literature is screened, literature titles and abstracts are screened for literature that does not meet the inclusion criteria, and finally the literature that meets the criteria is selected by reading the full text. If there are differences in the process of literature screening, consulting the relevant experts or discussing them with a third researcher. If several papers have been published in the same study, the literature with the most complete experimental data and the most consistent with the inclusion criteria is selected for inclusion in the study. All the selected literatures that met the inclusion criteria are sorted out and analyzed by office software: basic literature information (title, author, year of publication), general situation (patient age, gender, number of cases, etc.), experimental design (interventions), outcome indicators. If the outcome indicator units in the literature are not consistent, the units are converted uniformly and then subsequent data processing is carried out. If there is a lack of data in the literature, trying to contact the original author by e-mail or telephone and obtain the relevant information needed. The PRISMA flow chart of literature selection is shown in Figure 1.

### 2.8 Literature quality assessment

The selected literature conducts bias risk evaluation based on the method recommended by Coachrane Handbook for System Reviews of Interventions 5.1.0 and its results are reported. Evaluation items include: random grouping methods, allocation of hidden scheme design, use of blind methods, reporting of results data, whether there is any selective reporting of research results, whether there are other sources of bias, etc. The results are as follows: “Yes” represents that the method is correct or the data is complete, indicating that bias risk is small; “Unclear” represents that the method is not clear, indicating that bias risk is moderate; “No” represents that the method is incorrect or the data is incomplete, indicating that bias risk s is high. Finally, the evaluation results were input into RevMan 5.3 software, and the bias risk assessment chart is output.

### 2.9 Statistical method

Conducting statistical analysis by using the RevMan 5.3 software recommended by the Cochrane Collaboration Network. The statistics of counting data are expressed by odds ratio (OR), while statistics of measurement data are expressed by mean difference (MD). Both the effect quantity of counting data and the measurement data are expressed as 95% confidence interval (CI). For heterogeneity test, when the statistics *P* > .1, I^2^ < 50% can be considered to have a higher homogeneity between the results of the study, indicating that there is no significant statistical difference in the inclusion data, at this time, using the fixed effect model; when the statistic *P* ≤ .1, I^2^ ≥ 50% can be considered that there is heterogeneity between the results of the study, indicating that there is significant statistical differences in the inclusion data, and taking into account the factors that might cause heterogeneity, and that there might be heterogeneity. The factors of increased quality are analyzed in subgroups according to actual needs, and if subgroup analysis shows *P* ≤ .1, I^2^ ≥ 50%, the results is that there is heterogeneity, using random effect models. On the contrary, if there are statistical differences in the results of each subgroup, but there is no clinical heterogeneity, using the fixed effect model to conduct the meta-analysis, When the heterogeneity of the outcome index is too large, it can be considered that the actual need for descriptive analysis of relevant literature research. Sensitivity analysis is used to test whether the results are stable.^[14,15]^

#### 2.9.1 Dealing with missing data

In case of missing data or incomplete data reports, we will try to contact the corresponding authors to obtain the original data. If the author cannot be contacted, we will analyze it based on the available data.

#### 2.9.2 Sensitivity analysis

Sensitivity analysis: the implementation method is divided into change analysis model, one by one to remove the included research (deleting one by one a result indicator included in the study, observing whether the heterogeneity has changed, while recording the numerical changes of the combined effect value. If it is found that there is a significant change in the heterogeneity of the outcome indicator after the removal of a document, then the literature is the source of heterogeneity). When the conclusions are unified before and after the sensitivity analysis, the conclusions are stable, and when the conclusions are inconsistent before and after the sensitivity analysis, the conclusions are not stable and it is needed to be treated with caution.

#### 2.9.3 Subgroup analysis

This study will conduct a subgroup analysis of factors that have an effect on outcome indicators, including the age of the child, course of illness, dose of drug use, frequency of drug use and course of treatment.

### 2.10 Publication bias

Publication bias is identified by funnel plot. Funnel plot is a qualitative evaluation method, which can be visually identified by funnel pattern symmetry to determine whether the bias is exist. The included literature uses a funnel chart for publication bias analysis, if the two sides of the graph are basically symmetrical, then the bias is smaller, and conversely, the bias is larger.

### 2.11 Grading the quality of evidence

Based on the results of systematic evaluation, the quality of evidence is evaluated by GRADE, which is an internationally used evidence quality grading system, and the quality of evidence is graded as follows:

1. high quality: further research is unlikely to change the credibility of the results of the effectiveness assessment.
2. medium quality: further research is likely to affect the credibility of the results of the effectiveness assessment and it may change the results.
3. low quality: further research is likely to affect the credibility of the results of the effectiveness assessment, and the results are likely to change.
4. very low quality: the results of any effectiveness assessment are uncertain.

Recommendations are classified as “Strong” and “Weak”: strong recommendations indicate that the evaluator has a clear indication that the intervention has more advantages or disadvantages than benefits, and weak recommendations indicate that the intervention has more advantages or disadvantages than benefits. The included study is all randomized controlled trials, and although evidence is first classified as high quality based on randomized controlled trials, the quality of such evidence may be reduced by five factors: risk of research bias, discrete research results, indirect evidence, imprecise results, and publication bias.

## 3 Discussion

REC is the most common viral diarrhea in children, of which rotavirus is the most common pathogen of autumn and winter diarrhea in infants and young children. After infection, rotavirus invades the columnar epithelial cells at the top of the small intestinal microvilli, resulting in vacuolar degeneration and necrosis of the cells, water and electrolyte re-absorption function are impaired, a large amount of fluid is gathered in the intestinal cavity to cause diarrhea, In addition, the function of small intestinal mucosal epithelial cells is impaired, disaccharide enzyme secretion is reduced, activity is reduced, lactose is retained in the intestinal lumen, resulting in increased osmotic pressure and osmotic diarrhea. REC often combines multi-organ damage, which often accompanied by dehydration and electrolyte disorders, and it can lead to death in severe cases. Although the disease is a self-limiting disease, it is also a common disease and often has complications. Acute diarrhea can lead to dehydration symptoms, chronic diarrhea can cause weakened immunity, growth and developmental retardation, malnutrition, abnormal digestive function, etc., and become the leading cause of death. Therefore, for the disease, timely treatment is of great significance.^[16]^ As one of the most important means, drug treatment is the focus of people's focus.

Xingpi Yanger granule is a modern traditional Chinese medicine preparation. Its main components are *Emilia sonchifolia (L.) DC, Gerbera piloselloides (Linn.) Cass, Pittosporum illicioides Makino, Valeriana jatamansi Jones*, etc. It is a pure traditional Chinese medicine preparation. It can antagonize barium chloride, acetylcholine and histamine, significantly increase the activity of pepsin, increase the concentration and secretion of trypsin, relieve the inhibition of adrenaline, and has excellent effects of relieving spasm, pain and diarrhea. *Emilia sonchifolia (L.) DC* can clear heat and detoxify, eliminate inflammation and diuresis, treat dyspepsia, intestinal ulcers and other diseases. *Gerbera piloselloides (Linn.) Cass* can dry damp and strengthening spleen, treatment of children with abdominal distension, dyspepsia and dyspepsia. *Pittosporum illicioides Makino* can benefit *Qi*, nourish blood, calm and soothe the nerves. *Valeriana jatamansi Jones* can eliminate inflammation and diarrhea, regulate *Qi* and relieve pain, and treat dyspepsia, abdominal pain and diarrhea.^[17]^ Saccharomyces boulardii is a fungal micro-ecological preparation that temporarily acts as an intestinal probiotic, maintains the micro-ecological balance of the intestine, inhibits the reproduction of pathogenic microorganisms, neutralizes and degrades toxins, protects epithelial cells from invasion, and improves the metabolic function and nutritional function of the intestinal mucosa.^[18]^

Although XPYEG and SBI have been widely used in the treatment of REC, there are not many studies on the combination of drugs, the study quality is low, and the effectiveness and safety are also controversial. Therefore, this study intends to find high-quality evidence-based medical evidence for the treatment of REC with XPYEG combined with SBI by meta-analysis.

## Author contributions

**Conceptualization:** Cheng-Ying Qiu, Shan-Wen Huang.

**Data curation:** Cheng-Ying Qiu, Zao-Xia Guo, Gui-Hua Zhang, Yong-Hong Feng.

**Formal analysis:** Cheng-Ying Qiu, Zao-Xia Guo, Gui-Hua Zhang, Yong-Hong Feng.

**Funding acquisition:** Shan-Wen Huang.

**Methodology:** Cheng-Ying Qiu, Gui-Hua Zhang, Yong-Hong Feng, Ying-Yun Deng.

**Software:** Zao-Xia Guo, Xian-Jia Chen, Xiao-Dong Wu.

**Writing – original draft:** Cheng-Ying Qiu, Zao-Xia Guo, Gui-Hua Zhang, Yong-Hong Feng, Ying-Yun Deng, Xian-Jia Chen, Xiao-Dong Wu.

**Writing – review & editing:** Shan-Wen Huang.
